# Cord Blood 3-Epi-25-Hydroxyvitamin D_3_ and Autism-Related Cognitive Traits at Age 10 Years: An Exploratory Secondary Analysis of the KLOTHO Birth Cohort

**DOI:** 10.3390/nu18203361

**Published:** 2026-10-14

**Authors:** Spyridon N. Karras, Dimitrios G. Goulis, Maria Dalamaga, Maria Kypraiou, Antonios Vlastos, Marios Anemoulis, Vikentia Harizopoulou, Georgios Mastorakos, Dimitrios Skoutas, Stergios A. Polyzos, Antonios Goulas

**Affiliations:** 1 Laboratory of Pharmacology, Medical School, Aristotle University, 54124 Thessaloniki, Greece; spolyzos@auth.gr (S.A.P.); agoulas@auth.gr (A.G.); 2 Unit of Reproductive Endocrinology, 1st Department of Obstetrics and Gynecology, Medical School, Faculty of Health Sciences, Aristotle University, 54124 Thessaloniki, Greece; 3 Department of Biological Chemistry, National and Kapodistrian University of Athens, 15771 Athens, Greece; madalamaga@med.uoa.gr; 4 Assisting Nature Centre of Reproduction and Genetics, 57001 Thessaloniki, Greece; mariabioanalysis@yahoo.gr; 5 Medical School, Aristotle University, 54124 Thessaloniki, Greecemariosanemoulis@hotmail.com (M.A.); 6 Department of Midwifery, Faculty of Health and Caring Sciences, University of West Attica, 12243 Athens, Greece; vharizopoulou@uniwa.gr; 7 Second Department of Surgery, Medical School, Aretaieio Athens Hospital, National and Kapodistrian University of Athens, 11528 Athens, Greece; gmastorak@med.uoa.gr; 8 Thermi Clinic, 57001 Thessaloniki, Greece

**Keywords:** 3-epi-25-hydroxyvitamin D_3_, vitamin D metabolism, cord blood, prenatal exposure, neurodevelopment, autism-related traits, birth cohort, child development

## Abstract

Background: The C3 epimer of 25-hydroxyvitamin D_3_ [3-epi-25(OH)D_3_] can be measured in maternal and umbilical cord circulation, but whether it is related to later neurodevelopment is unknown. Objective: To explore associations between cord 3-epi-25(OH)D_3_ and parent-reported autism-related trait domains at 10 years in the KLOTHO birth cohort. Methods: This secondary analysis used prospectively collected perinatal samples and 10-year questionnaire data. Vitamin D metabolites were quantified by liquid chromatography–tandem mass spectrometry with epimer separation. The Australian Scale for Asperger’s Syndrome was used dimensionally, not diagnostically. Spearman correlations, false-discovery-rate (FDR) correction across 13 unique bivariate comparisons, supportive linear regressions, sex-stratified analyses, and a stricter record-linkage sensitivity analysis were conducted. Results: Data were available for 87 offspring (41 boys and 46 girls). Cord 3-epi-25(OH)D_3_ was inversely associated with cognitive-domain score (Spearman ρ = −0.412; *p* < 0.001; Benjamini–Hochberg q = 0.001). The crude regression estimate was also inverse (B = −0.285; 95% CI, −0.458 to −0.112; *p* = 0.002), but the association attenuated after covariate adjustment and all adjusted confidence intervals included the null. A stricter linkage analysis remained significant (n = 84; ρ = −0.350; *p* = 0.001). The correlation was numerically stronger in boys than girls, but the between-sex comparison was not significant (*p* = 0.075). A positive correlation was also observed for social and emotional traits (ρ = 0.302; q = 0.023), indicating inconsistent directions across domains; the tertile analysis was non-significant. Conclusions: Cord 3-epi-25(OH)D_3_ was associated with lower cognitive-domain scores in unadjusted rank analyses, but the lack of stable adjusted linear evidence, inconsistent domain pattern, and small sample restrict interpretation. These findings are hypothesis-generating and do not imply causality, sex-specific effects, or clinical utility.

## 1. Introduction

Autism-related characteristics are distributed dimensionally across the population and include variations in social interaction, communication, restricted interests, and features captured by cognitive and motor screening domains. They arise from interacting genetic and environmental determinants across prenatal and postnatal development [1]. The vitamin D receptor (VDR) and enzymes involved in vitamin D activation and catabolism have been demonstrated in neural tissues, providing a plausible, but non-specific, biological context for effects on neuronal differentiation, neurotransmitter signaling, synaptic maturation, and neuroimmune processes [2,3]. These pathways could be relevant to later performance or behavior measured by developmental instruments; however, neither the presence of VDR nor experimental vitamin D signaling establishes that a particular circulating vitamin D epimer directly affects cognitive traits.

Human data on maternal and neonatal vitamin D concentrations and subsequent neurodevelopment are mixed. Observational studies have reported associations with language, cognition, attention, behavior, or autism-related outcomes, whereas other cohorts and genetically informed analyses have found weak or null associations after adjustment [4,5,6,7,8,9,10,11,12,13,14,15,16]. A randomized trial following children to age 10 years found associations of pre-intervention maternal 25(OH)D with some outcomes, but no benefit of high-dose versus standard-dose prenatal vitamin D_3_ supplementation on autism or ADHD diagnoses [17]. Recent systematic syntheses report population-level associations between prenatal vitamin D status and autism-related outcomes, but differences in exposure measurement, confounding, and outcome definitions continue to limit causal inference [18,19]. Thus, a maternal vitamin D signal should not automatically be interpreted as evidence for a preventive intervention or for a direct effect of a specific metabolite.

Most epidemiological studies measure conventional 25-hydroxyvitamin D [25(OH)D] or a total 25(OH)D value. The C3 epimer 3-epi-25(OH)D_3_ differs in stereochemistry from 25(OH)D_3_ and may co-elute in assays without suitable chromatographic separation [20,21,22,23,24,25,26]. Epimer concentrations are measurable in maternal, cord, and infant circulation and vary over the perinatal period. Studies of paired mother–infant samples suggest that fetal–placental synthesis or handling, rather than a simple passive transplacental transfer of an unchanged maternal epimer pool, may contribute to cord concentrations [22,24]. This is important analytically because non-separated measurements may misclassify conventional vitamin D status, and conceptually because the epimer may represent a different aspect of perinatal metabolism.

The maternal–placental–fetal axis provides the main rationale for an epimer-specific longitudinal question. Maternal substrate availability, supplementation, season, gestational physiology, and placental transport and enzymatic processes jointly influence the vitamin D metabolites measurable at delivery [22,24,27,28]. Cord 3-epi-25(OH)D_3_ could therefore serve as a proxy for metabolic exposures during fetal development, rather than acting as an established neuroactive molecule. Because aspects of neural differentiation, synaptic organization, and neuroimmune development begin before birth, such an exposure marker might be statistically related to later developmental traits. This proposed pathway is indirect and remains untested; neither epimer concentrations nor domain scores can establish placental mediation or a direct neurobiological mechanism. The specific knowledge gap is whether analytically separated cord 3-epi-25(OH)D_3_ relates prospectively to later dimensional neurodevelopmental traits, beyond what has been examined for total 25(OH)D. We therefore performed an exploratory, metabolite-specific secondary analysis of the KLOTHO cohort, considering maternal and cord vitamin D metabolites and multiple parent-reported autism-related trait domains at age 10 years. The cognitive-domain association is presented as the focal observed signal; it should not be understood as a prospectively registered confirmatory primary outcome. We examined the multiplicity of correlations, consistency with adjusted regression, sensitivity to record linkage, and possible differences by sex without assuming a priori that a sex-specific effect existed.

## 2. Materials and Methods

### 2.1. Study Design and Participants

KLOTHO is a prospective mother–child birth cohort established in Thessaloniki, Greece. Pregnant women were recruited at the Maternity Unit of the First Department of Obstetrics and Gynecology, Aristotle University of Thessaloniki, during 2011. Maternal blood was obtained at delivery and umbilical cord blood immediately after birth. The cohort design and 10-year neurodevelopmental follow-up have been described previously [13,20].

**Figure 1:**
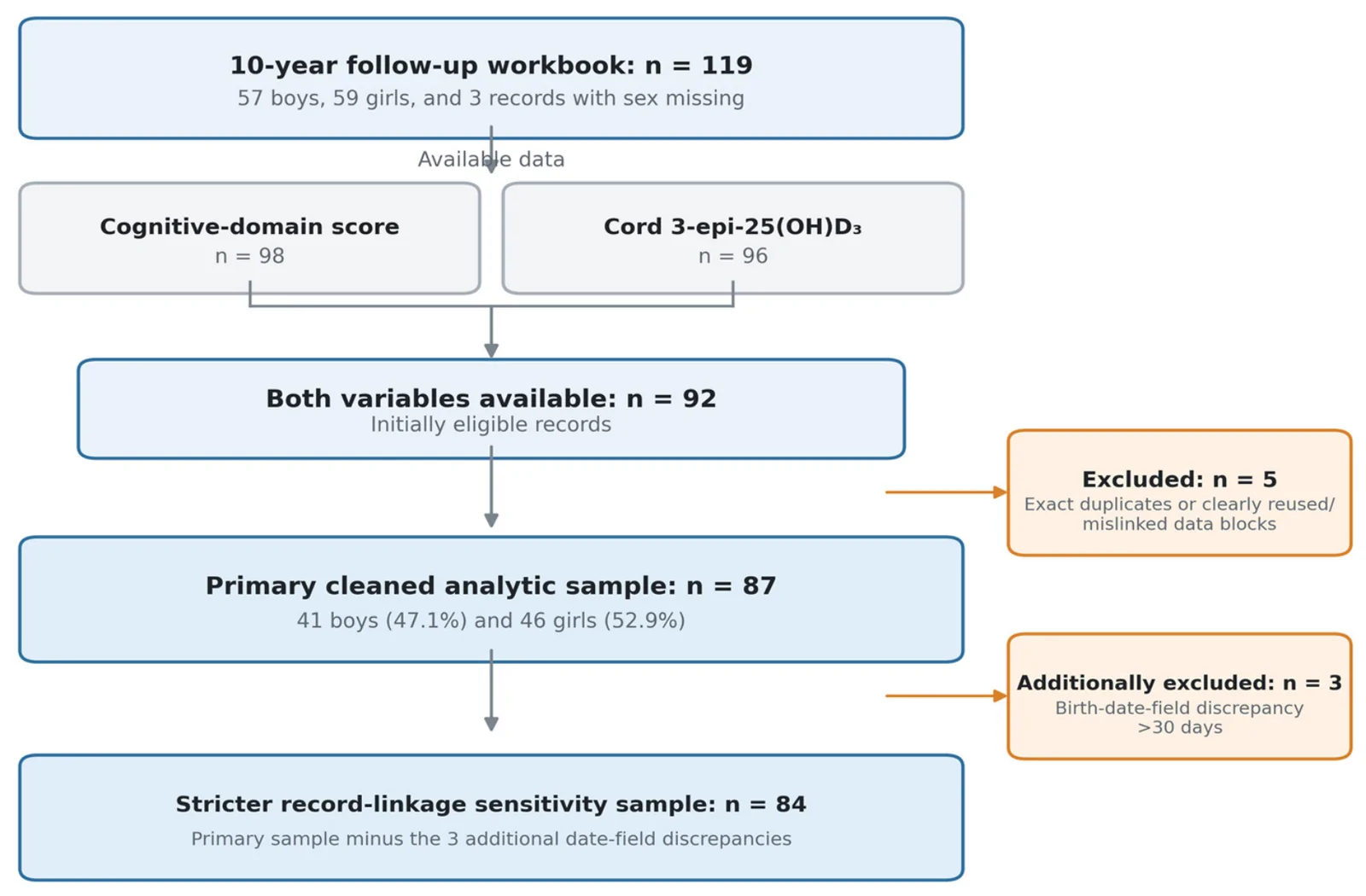
Flow of records from the 10-year follow-up workbook to the primary cleaned analytic sample and the stricter record-linkage sensitivity sample.

The 10-year follow-up workbook contained 119 records. Cognitive-domain data were available for 98 records, and cord 3-epi-25(OH)D_3_ measurements for 96; a total of 92 records had both measurements. A record-integrity review compared repeated perinatal and follow-up data patterns and the consistency of available linkage fields. Five records were removed because they were exact duplicates or showed an objectively repeated perinatal or follow-up data block that was inconsistent with the linked record. The primary analytic sample included 87 offspring (41 boys and 46 girls). Three additional records had discrepancies greater than 30 days between two recorded birth-date fields. Unlike the five excluded records, these date discrepancies alone did not establish duplication or an inconsistent data block; the three records were therefore retained in the primary analysis and excluded only in a stricter record-linkage sensitivity analysis (n = 84). No missing data were imputed. Figure 1 summarizes participant selection.

### 2.2. Ethics

This study was conducted in accordance with the Declaration of Helsinki and approved by the Institutional Review Board of the Medical School of Aristotle University of Thessaloniki (approval no. 1/19-12-2011; 1 February 2012). Informed consent was obtained from all participants involved in the study, including consent for offspring follow-up.

### 2.3. Vitamin D Metabolite Assessment

Maternal serum and umbilical cord serum were processed and stored at −20 °C until analysis, according to the standardized cohort protocol previously described [20]. Concentrations of 25(OH)D_3_, 3-epi-25(OH)D_3_, and 25(OH)D_2_ were quantified using a liquid chromatography–tandem mass spectrometry method capable of chromatographic separation of the C3 epimer. Total 25(OH)D was derived from the measured vitamin D forms. The assay approach and the maternal–neonatal metabolite protocol have been reported previously [20]. The focal exposure in the present analysis was cord blood 3-epi-25(OH)D_3_, expressed in nmol/L.

### 2.4. Autism-Related Trait Assessment

Parents completed the Australian Scale for Asperger’s Syndrome at follow-up [29]. The instrument is a parent-rated screening questionnaire designed for children in the primary-school years and contains 24 scored items across five domains: social and emotional abilities (10 items; possible score 0–60), communication skills (6 items; 0–36), cognitive skills (3 items; 0–18), specific interests (3 items; 0–18), and movement skills (2 items; 0–12). Each item is rated from 0, representing the ordinary level expected for a child of that age, to 6, representing frequent or marked expression of the characteristic. Domain scores were calculated by summing the relevant items, and the composite score was calculated as the sum of the 24 scored items (possible range 0–144), according to the cohort scoring protocol. Higher scores indicate more autism-related characteristics. No item-level imputation was performed; domain-specific analyses included participants with an available calculated score for the corresponding domain. The cognitive subscale contains only three parent-reported items (range, 0–18) and should not be interpreted as an objective or comprehensive measure of cognitive function. The instrument was used dimensionally as a screening measure and not to establish an autism spectrum disorder diagnosis. The neurodevelopmental questionnaires used in the KLOTHO follow-up have previously been reported to show acceptable internal consistency and construct validity in Greek populations [13].

### 2.5. Covariates

The available covariates considered in supportive regression models were offspring sex, maternal educational level, season of delivery, and estimated ultraviolet-B (UVB) exposure. Models were kept parsimonious given the limited analytic sample. Maternal vitamin D supplementation was not included in the reported models. Because supplementation may influence vitamin D metabolites and correlate with nutritional, socioeconomic, and healthcare-related characteristics, this omission constitutes a potential source of residual confounding. Other potentially relevant factors not comprehensively controlled included maternal age, prepregnancy adiposity, gestational weight gain, smoking, alcohol consumption, diet, parity, maternal mental health, pregnancy complications, gestational diabetes, hypertensive disorders, gestational age, socioeconomic status, and paternal characteristics. Complete parental-age information was not available across the analytic sample. Consequently, the adjusted models should be viewed as supportive rather than fully confounder-adjusted estimates. Variable-specific sample sizes for available descriptive data are reported in Table 1; no imputation was performed.

### 2.6. Statistical Analysis

Continuous variables were summarized using the mean and standard deviation (SD) when approximately symmetric and the median and interquartile range (IQR) when skewed. Ranges were not routinely reported. Spearman rank correlations were used because epimer concentrations were right-skewed and included a high value. Approximate 95% confidence intervals for Spearman coefficients were calculated using Fisher’s z transformation, and two-sided *p*-values were calculated using the corresponding t approximation with n − 2 degrees of freedom.

Thirteen unique bivariate tests were evaluated: eight maternal or cord vitamin D metabolite correlations with the cognitive-domain score, plus five additional domain correlations with cord 3-epi-25(OH)D_3_ (the cord epimer–cognitive association occurs in two descriptive tables but was counted only once). Nominal *p*-values are presented alongside Benjamini–Hochberg false-discovery-rate q-values calculated over this defined family of tests [30]. The FDR-controlled family was limited to these 13 unique bivariate correlations; sex-stratified correlations, tests of between-sex differences, ordinary least-squares regressions, tertile comparisons, and record-linkage sensitivity analyses were supplementary exploratory assessments and were not part of this FDR correction. Their unadjusted *p*-values must not be interpreted as confirmatory evidence. Because the analyses are exploratory, an FDR-adjusted association indicates a relatively low expected proportion of false discoveries within the selected family under the method’s assumptions; it does not establish independence from confounding or eliminate post hoc selection of the focal domain.

Sex-stratified Spearman coefficients were calculated separately for boys and girls, and their difference was tested directly using Fisher’s z approximation. Supportive ordinary least-squares models estimated unstandardized coefficients (B), representing cognitive-domain score points per 1 nmol/L higher cord 3-epi-25(OH)D_3_ concentration. Conventional model-based standard errors and 95% confidence intervals were calculated. Models included the unadjusted association and separately specified sparse adjustments for offspring sex, maternal educational level, season, and UVB exposure as available; these should not be interpreted as a single fully adjusted causal model. Cord epimer tertiles were assessed with the Kruskal–Wallis test. The outcome is a bounded 0–18 score based on three ordinal items, while the exposure is right-skewed and contains a high observation area; ordinary least-squares assumptions concerning linearity, residual behavior, and sensitivity to leverage may therefore be violated. The OLS models are supportive descriptive analyses, not definitive independent or causal estimates. Small subgroup sizes, residual confounding, and possible non-linearity were considered during interpretation. No causal identification strategy or confirmatory primary outcome was defined for this secondary analysis. Statistical computations were performed using IBM SPSS Statistics (IBM Corp., Armonk, NY, USA), and FDR and between-sex calculations were independently checked from the reported summary statistics.

The stricter record-linkage sensitivity analysis retained 84 of the 87 primary records, excluding the three observations with unresolved birth-date discrepancies described above. No missing values were imputed.

## 3. Results

### 3.1. Analytic Sample

The primary cleaned analytic sample included 87 offspring. Cord 3-epi-25(OH)D_3_ was right-skewed, with a median of 1.72 nmol/L (IQR, 0.50–3.65). The cognitive-domain score had a median of 5.00 (IQR, 0.00–6.00). The available descriptive characteristics are presented with variable-specific denominators in Table 1.

### 3.2. Vitamin D Metabolites and Cognitive-Domain Score

Cord 3-epi-25(OH)D_3_ had the largest absolute correlation with the cognitive-domain score (ρ = −0.412; *p* < 0.001; q = 0.001). Maternal 25(OH)D_2_ also showed an inverse correlation (ρ = −0.296; *p* = 0.005; q = 0.023). The remaining maternal and cord metabolite correlations did not meet the 5% false-discovery-rate threshold (Table 2).

**Table 2:** Maternal and cord vitamin D metabolites in relation to cognitive-domain score.

Vitamin D Metabolite	n	Spearman ρ	Nominal *p*	BH q
Maternal 25(OH)D_3_	87	0.170	0.115	0.312
Maternal 3-epi-25(OH)D_3_	87	0.005	0.963	0.963
Maternal 25(OH)D_2_	87	−0.296	0.005	0.023
Maternal total 25(OH)D	87	0.020	0.855	0.963
Cord 25(OH)D_3_	87	0.168	0.120	0.312
Cord 3-epi-25(OH)D_3_	87	−0.412	<0.001	0.001
Cord 25(OH)D_2_	87	0.027	0.804	0.963
Cord total 25(OH)D	86	0.040	0.715	0.963

BH q-values were calculated across 13 unique bivariate tests reported in Table 2 and Table 3; the cord epimer–cognitive test was counted once. BH, Benjamini–Hochberg.

**Table 3:** Cord 3-epi-25(OH)D_3_ in relation to autism-related trait domains.

Outcome	n	Spearman ρ	Nominal *p*	BH q
Social and emotional traits	87	0.302	0.004	0.023
Communication traits	87	−0.013	0.905	0.963
Cognitive traits	87	−0.412	<0.001	0.001
Special interests	87	−0.133	0.219	0.421
Motor characteristics	87	0.131	0.226	0.421
Composite trait score	87	0.066	0.544	0.883

BH q-values were calculated across the 13 unique bivariate tests reported in Table 2 and Table 3; the cord epimer–cognitive test was counted once.

### 3.3. Cord Epimer and Autism-Related Trait Domains

The largest absolute domain-specific estimate was the inverse correlation with the cognitive score (ρ = −0.412; *p* < 0.001; q = 0.001). The correlation with the social and emotional score was positive (ρ = 0.302; *p* = 0.004; q = 0.023). Estimates for communication, special interests, motor characteristics, and the composite score were smaller and did not meet the false-discovery-rate threshold (Table 3).

### 3.4. Sex-Stratified Estimates

**Figure 2:**
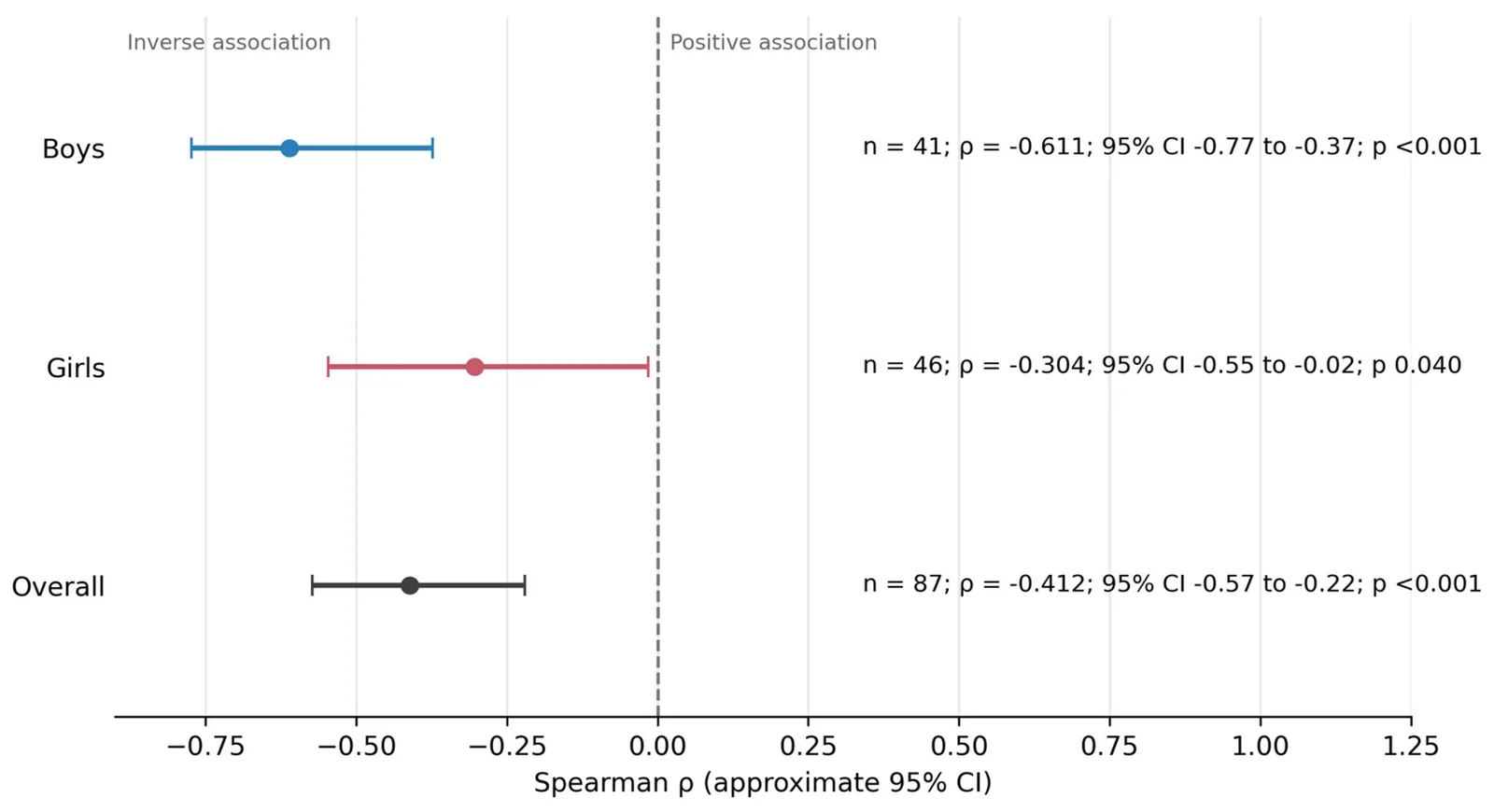
Sex-stratified associations of cord blood 3-epi-25(OH)D_3_ with cognitive-domain score. Points show the Spearman coefficients and horizontal lines show approximate 95% confidence intervals. The formal boys-versus-girls comparison was non-significant (Fisher z = −1.78; *p* = 0.075).

The inverse correlation was larger in boys (ρ = −0.611; *p* < 0.001) than in girls (ρ = −0.304; *p* = 0.040). However, the formal between-sex comparison was not statistically significant (Fisher z = −1.78; *p* = 0.075). Thus, the data support a larger point estimate in boys but do not establish effect modification by sex (Table 4). The sex-stratified coefficients and their approximate 95% confidence intervals are shown in Figure 2.

Confidence intervals use Fisher’s z approximation. Boys versus girls: Fisher z = −1.78, *p* = 0.075.

### 3.5. Regression and Tertile Analyses

**Figure 3:**
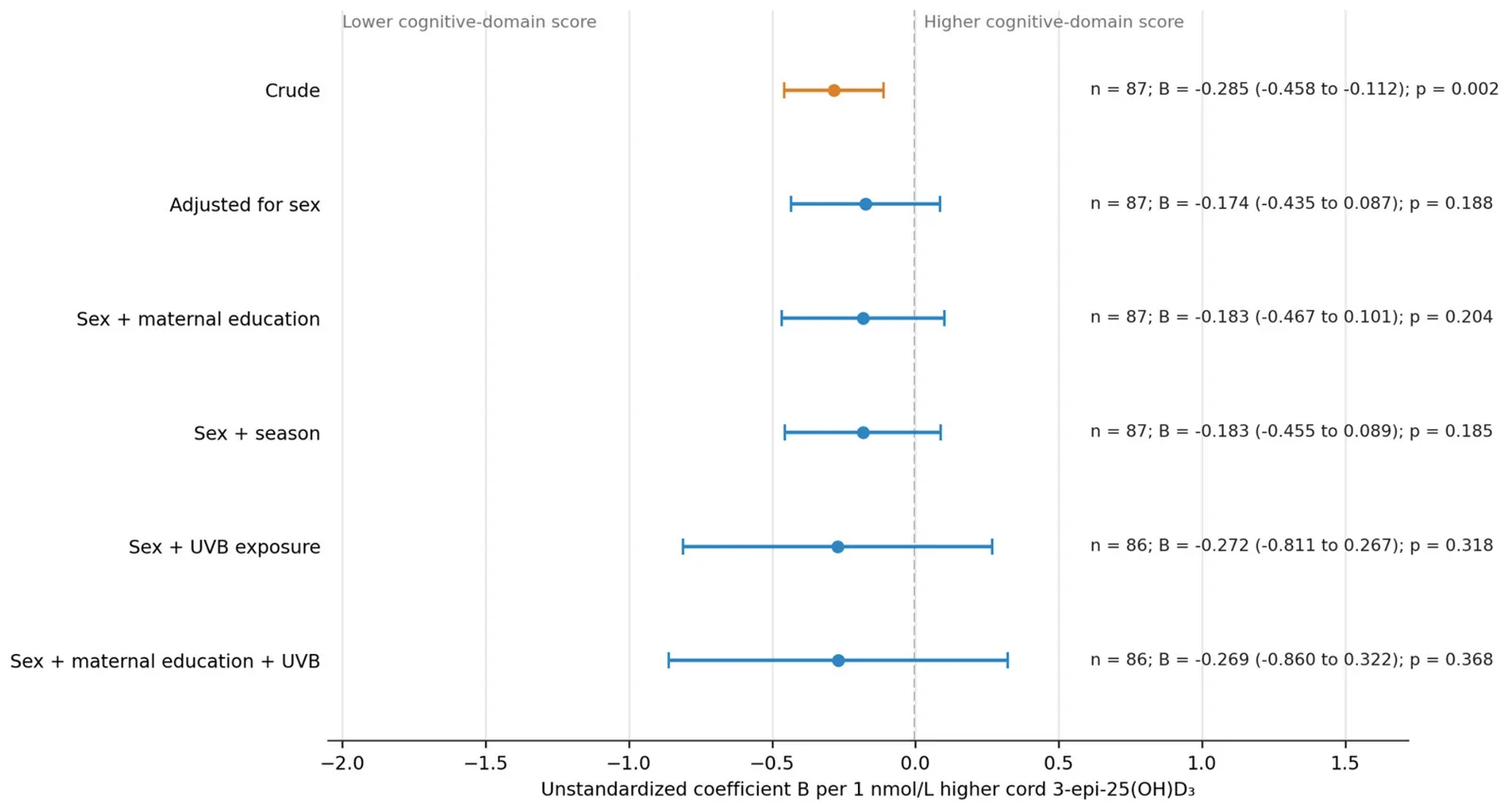
Crude and adjusted linear associations between cord blood 3-epi-25(OH)D_3_ and the cognitive-domain score. Points represent unstandardized regression coefficients (B) per 1 nmol/L higher cord 3-epi-25(OH)D_3_ concentration, and horizontal lines represent 95% confidence intervals. The dashed vertical line denotes the null value. Only the crude-model confidence interval excluded the null.

In the crude model, a 1 nmol/L higher cord 3-epi-25(OH)D_3_ concentration corresponded to a 0.285-point lower cognitive-domain score (B = −0.285; SE = 0.087; 95% CI, −0.458 to −0.112; *p* = 0.002). The estimate attenuated after adjustment for sex (B = −0.174; *p* = 0.188), sex and maternal education (B = −0.183; *p* = 0.204), or sex and season (B = −0.183; *p* = 0.185). The models also including UVB had confidence intervals crossing zero (Table 5, Figure 3). Thus, the inverse rank association did not translate into a consistently significant covariate-adjusted linear effect, a discrepancy that is central to interpretation and not resolved by FDR correction of bivariate tests.

In the boys-only models, unstandardized coefficients ranged from −1.040 to −1.126 points per 1 nmol/L higher cord epimer concentration and remained nominally significant in the crude model and after separate adjustment for maternal education or season (Table 6). These estimates are based on 41 boys and should be regarded as exploratory. In girls, the corresponding rank association was weaker (ρ = −0.304; *p* = 0.040), and the formal boys-versus-girls comparison was not statistically significant; therefore, the boys-only linear models should not be interpreted as evidence of sex-specific effect modification.

Across cord epimer tertiles, the mean cognitive-domain scores were 5.31 ± 3.15 in the lowest tertile, 4.38 ± 4.24 in the middle tertile, and 3.09 ± 3.48 in the highest tertile. The overall difference was not significant (Kruskal–Wallis H = 2.745; *p* = 0.253), providing no clear evidence of a monotonic tertile pattern. In the stricter record-linkage sensitivity analysis (n = 84), the focal rank correlation was smaller in magnitude but remained statistically significant (ρ = −0.350; *p* = 0.001).

## 4. Discussion

This exploratory analysis of the KLOTHO cohort identified an inverse rank association between cord 3-epi-25(OH)D_3_ and the cognitive domain of parent-reported autism-related traits at age 10 years. The Spearman association remained significant after FDR correction across 13 unique comparisons and after stricter record-linkage screening. Nevertheless, the initially significant crude linear estimate weakened after adjustment; no adjusted 95% confidence interval excluded zero. The tertile analysis was non-significant, correlations differed in direction across domains, and the direct between-sex contrast was also non-significant. Taken together, these patterns do not demonstrate a stable independent linear dose–response relationship.

The findings extend the previously reported KLOTHO investigation of conventional maternal and neonatal vitamin D status and neurodevelopment [13]. Whereas the earlier report analyzed 66 mother–child pairs for its specified endpoints, the present secondary analysis includes 87 offspring with cord epimer and domain-specific questionnaire data. These analytic samples represent different combinations of available exposure and outcome measurements and should not be interpreted as directly interchangeable. Moreover, the inverse correlation between maternal 25(OH)D_2_ and cognitive-domain score within the present family of tests argues against assuming that the observed association is specific to the cord C3 epimer.

The broader literature is neither uniformly null nor causally conclusive. Recent systematic and dose–response meta-analyses report inverse population-level associations between maternal 25(OH)D and offspring autism-related outcomes, while also emphasizing heterogeneity and possible residual confounding [18,19]. In contrast, a randomized comparison of high- versus standard-dose prenatal vitamin D_3_ did not reduce autism or ADHD diagnoses at 10 years, despite associations with baseline maternal vitamin D status [17]. These studies concern conventional vitamin D measures and clinical or behavioral outcomes, not the C3 epimer; they support contextual plausibility but cannot validate the present metabolite-specific signal.

Chromatographic separation is a major strength because 3-epi-25(OH)D_3_ can otherwise contribute to measured 25(OH)D_3_ concentrations and distort interpretation in pregnancy and infancy [21,22,23,24,25,26]. However, the C3 epimer is not established as a neurodevelopmentally active ligand at the concentrations studied. Maternal nutritional and metabolic exposures, changing gestational physiology, and placental transport and enzymatic activity can all influence the cord epimer pool [22,24,27,28]. Its association with a later score might consequently identify a fetal–placental metabolic phenotype rather than direct epimer neuroactivity; no placental, neural, or mechanistic intermediate was measured here.

Several features caution against overinterpretation. The epimer distribution was right-skewed with a high value, potentially affecting ordinary least-squares leverage and functional-form assumptions. Even a modest adjustment attenuated the coefficient, and a comparison of tertiles did not show a statistically reliable gradient. Spearman’s correlation is a useful rank-based summary, but it neither controls for measured or unmeasured confounders nor guarantees a linear association. Moreover, the selected FDR family addresses multiple testing within the reported comparisons, not all possible decisions made during the exploratory analytic process.

The trait-domain results were not uniformly favorable: a higher cord epimer concentration correlated with lower cognitive-domain scores (ρ = −0.412) but with higher social and emotional scores (ρ = 0.302), and both survived the specified FDR correction. Because higher scores on this questionnaire denote more autism-related characteristics, the second association points in the opposite interpretative direction. Most other domains and the composite score did not show significant associations. It would therefore be inappropriate to label higher epimer concentrations globally protective or to equate questionnaire domains with clinically diagnosed autism.

The inverse correlation was numerically stronger in boys, but the formal between-sex test was not significant (Fisher z = −1.78; *p* = 0.075). Significant within-boy analyses and a larger point estimate are insufficient to establish an interaction. Sex-stratified regressions based on only 41 boys are vulnerable to instability, and the current data do not support a sex-specific interpretation.

Clinical inference is correspondingly limited. The study does not warrant altering recommended prenatal vitamin D testing or supplementation, targeting the C3 epimer, or using cord epimer measurements to predict neurodevelopment. Replication of the rank association in adequately powered independent samples, and demonstration that it persists under better confounder control, are prerequisites to any clinical consideration [27,31,32].

### Strengths and Limitations

The strengths include the longitudinal mother–child cohort, delivery-time sampling, epimer-resolving LC-MS/MS, a 10-year follow-up, reporting across multiple outcomes, FDR correction, a direct between-sex comparison, and explicit record-linkage sensitivity analyses.

This study has several limitations. The focal sample was modest (n = 87), particularly for sex-specific and covariate-adjusted models, and selection related to stored samples or follow-up participation cannot be excluded. Five records were excluded because of duplicates or incompatible linked data blocks, and three further records with discrepant birth-date entries were omitted from the stricter sensitivity analysis; residual record-linkage uncertainty remains possible. The cognitive domain of the Australian Scale for Asperger’s Syndrome comprises only three parent-reported items and must not be interpreted as an objective or comprehensive assessment of cognitive functioning. Maternal vitamin D supplementation, dietary intake, prepregnancy adiposity, pregnancy complications, socioeconomic factors, and parental neurodevelopmental or psychiatric characteristics were not comprehensively accounted for. Furthermore, the bounded cognitive-domain score and right-skewed epimer concentrations, including a high exposure value, may challenge assumptions of ordinary least-squares regression. The absence of a significant tertile trend, the attenuation of estimates after covariate adjustment, and the opposite directions of association across domains reduce confidence in a stable linear dose–response relationship. Together with the exploratory secondary design, these limitations preclude causal and clinical conclusions.

Future independent mother–child cohorts should prespecify the C3 epimer exposure, metabolite-specific hypotheses, and neurodevelopmental endpoints. Standardized LC-MS/MS or equivalent epimer-resolving methods, joint maternal and cord sampling, and repeated measurements during pregnancy and early infancy could distinguish maternal substrate availability from placental and fetal metabolism. Analyses should capture supplementation, dietary vitamin D, season, maternal metabolic health, gestational complications, socioeconomic factors, parental age and psychiatric history, and relevant genetic determinants. Blinded validated multidimensional assessments, neuropsychological testing and, where indicated, diagnostic evaluation should complement parent reports. Larger samples are needed for prespecified interaction analyses, robust regression, potential nonlinear effects, and the replication of record-linkage decisions. Mechanistic studies could investigate placental epimer handling before any clinical interpretation is contemplated.

## 5. Conclusions

In the KLOTHO cohort, higher cord 3-epi-25(OH)D_3_ concentrations correlated with lower parent-reported cognitive-domain scores at age 10 years in unadjusted rank analyses; this association survived FDR correction and stricter record-linkage screening. However, adjusted linear estimates included the null, the tertile analysis was negative, trait-domain directions were inconsistent, and a sex interaction was not established. These exploratory data do not demonstrate a causal or clinically actionable relation and warrant independent replication with prespecified analyses and more comprehensive confounder control.

## Figures and Tables

**Table 1:** Analytic sample characteristics.

Characteristic	Value
Cord epimer and cognitive-domain data	n = 87
Boys	41 (47.1%)
Girls	46 (52.9%)
Cord 3-epi-25(OH)D_3_, median (IQR), nmol/L	1.72 (0.50–3.65)
Cognitive-domain score, median (IQR)	5.00 (0.00–6.00)
Maternal age at delivery, mean ± SD, years (n = 87)	33.81 ± 4.96
Maternal educational level (n = 87)	Elementary 45 (51.7%); secondary 40 (46.0%); higher 2 (2.3%)
Gestational age, mean ± SD, weeks (n = 87)	38.34 ± 1.20
Birth weight, mean ± SD, g (n = 87)	3355 ± 379
Season of delivery (n = 87)	September–March 75 (86.5%); April–May 12 (13.5%)
UVB exposure over preceding 45 days, mean ± SD, Wh/m^2^	0.229 ± 0.098
Child age at follow-up	10.47 ± 0.67 years

Values are presented as n (%) or mean ± standard deviation (SD), unless otherwise indicated. Percentages were calculated using the corresponding non-missing denominators. The descriptive statistics were verified against the final analytical dataset. No imputation of missing values was performed.

**Table 4:** Sex-stratified association of cord 3-epi-25(OH)D_3_ with cognitive-domain score.

Group	n	Spearman ρ	Approximate 95% CI	Nominal *p*
Boys	41	−0.611	−0.77 to −0.37	<0.001
Girls	46	−0.304	−0.55 to −0.02	0.040
Overall	87	−0.412	−0.57 to −0.22	<0.001

**Table 5:** Ordinary least-squares models for cognitive-domain score in the full sample.

Model	n	B	SE	95% CI	*p*
Crude	87	−0.285	0.087	−0.458 to −0.112	0.002
Sex	87	−0.174	0.131	−0.435 to 0.087	0.188
Sex and maternal education	87	−0.183	0.143	−0.467 to 0.101	0.204
Sex and season	87	−0.183	0.137	−0.455 to 0.089	0.185
Sex and UVB exposure	87	−0.272	0.271	−0.811 to 0.267	0.318
Sex, maternal education, and UVB	87	−0.269	0.297	−0.860 to 0.322	0.368

B is the unstandardized coefficient for cord 3-epi-25(OH)D_3_ and represents cognitive-domain score points per 1 nmol/L higher concentration. Conventional model-based standard errors and 95% confidence intervals are shown. UVB, ultraviolet B.

**Table 6:** Boys-only ordinary least-squares models for cognitive-domain score.

Model	n	B	SE	95% CI	*p*
Crude	41	−1.126	0.364	−1.862 to −0.390	0.004
Maternal education	41	−1.119	0.367	−1.862 to −0.376	0.004
Season	41	−1.040	0.420	−1.890 to −0.190	0.018

B is the unstandardized coefficient for cord 3-epi-25(OH)D_3_. The models are exploratory and not evidence of sex interaction.

## Data Availability

The original contributions presented in this study are included in the article. Further inquiries can be directed to the corresponding author.

## References

[1] Lord C., Brugha T.S., Charman T., Cusack J., Dumas G., Frazier T., Jones E.J., Jones R.M., Pickles A., State M.W. (2020). Autism spectrum disorder. Nat. Rev. Dis. Primers.

[2] Eyles D.W., Burne T.H., McGrath J.J. (2013). Vitamin D, effects on brain development, adult brain function and the links to neuropsychiatric disease. Front. Neuroendocrinol..

[3] Groves N.J., McGrath J.J., Burne T.H. (2014). Vitamin D as a neurosteroid affecting the developing and adult brain. Annu. Rev. Nutr..

[4] Whitehouse A.J.O., Holt B.J., Serralha M., Holt P.G., Kusel M.M.H., Hart P.H. (2012). Maternal serum vitamin D levels during pregnancy and offspring neurocognitive development. Pediatrics.

[5] Vinkhuyzen A.A.E., Eyles D.W., Burne T.H.J., E Blanken L.M., Kruithof C.J., Verhulst F., Jaddoe V.W., Tiemeier H., McGrath J.J. (2018). Gestational vitamin D deficiency and autism-related traits: The Generation R Study. Mol. Psychiatry.

[6] Morales E., Guxens M., Llop S., Rodríguez-Bernal C.L., Tardón A., Riaño I., Ibarluzea J., Lertxundi N., Espada M., Rodriguez A. (2012). Circulating 25-hydroxyvitamin D_3_ in pregnancy and infant neuropsychological development. Pediatrics.

[7] Daraki V., Roumeliotaki T., Koutra K., Chalkiadaki G., Katrinaki M., Kyriklaki A., Kampouri M., Margetaki K., Vafeiadi M., Papavasiliou S. (2018). High maternal vitamin D levels in early pregnancy may protect against behavioral difficulties at preschool age: The Rhea mother–child cohort, Crete, Greece. Eur. Child Adolesc. Psychiatry.

[8] Morales E., Julvez J., Torrent M., Ballester F., Rodríguez-Bernal C.L., Andiarena A., Vegas O., Castilla A.M., Rodriguez-Dehli C., Tardón A. (2015). Vitamin D in pregnancy and attention deficit hyperactivity disorder-like symptoms in childhood. Epidemiology.

[9] Vinkhuyzen A.A.E., Eyles D.W., Burne T.H.J., Blanken L.M.E., Kruithof C.J., Verhulst F., White T., Jaddoe V.W., Tiemeier H., McGrath J.J. (2017). Gestational vitamin D deficiency and autism spectrum disorder. BJPsych Open.

[10] Sourander A., Upadhyaya S., Surcel H.M., Hinkka-Yli-Salomäki S., Cheslack-Postava K., Silwal S., Sucksdorff M., McKeague I.W., Brown A.S. (2021). Maternal vitamin D levels during pregnancy and offspring autism spectrum disorder. Biol. Psychiatry.

[11] Lee B.K., Eyles D.W., Magnusson C., Newschaffer C.J., McGrath J.J., Kvaskoff D., Ko P., Dalman C., Karlsson H., Gardner R.M. (2021). Developmental vitamin D and autism spectrum disorders: Findings from the Stockholm Youth Cohort. Mol. Psychiatry.

[12] Schmidt R.J., Niu Q., Eyles D.W., Hansen R.L., Iosif A.M. (2019). Neonatal vitamin D status in relation to autism spectrum disorder and developmental delay in the CHARGE case-control study. Autism Res..

[13] Karras S.N., Goulis D.G., Kypraiou M., Harizopoulou V., Vlastos A., Anemoulis M., Tzimagiorgis G., Dalamaga M., Georgopoulos N., Kassi E. (2026). The KLOTHO Birth Cohort: Maternal and neonatal vitamin D status and neurodevelopmental outcomes at 10 years. Nutrients.

[14] Keim S.A., Bodnar L.M., Klebanoff M.A. (2014). Maternal and cord blood 25(OH)-vitamin D concentrations in relation to child development and behaviour. Paediatr. Perinat. Epidemiol..

[15] Darling A.L., Rayman M.P., Steer C.D., Golding J., Lanham-New S.A., Bath S.C. (2017). Association between maternal vitamin D status in pregnancy and neurodevelopmental outcomes in childhood: Results from the Avon Longitudinal Study of Parents and Children (ALSPAC). Br. J. Nutr..

[16] Madley-Dowd P., Dardani C., Wootton R.E., Dack K., Palmer T., Thurston R., Havdahl A., Golding J., Lawlor D., Rai D. (2022). Maternal vitamin D during pregnancy and offspring autism and autism-associated traits: A prospective cohort study. Mol. Autism.

[17] Aagaard K., Jepsen J.R.M., Sevelsted A., Horner D., Vinding R., Rosenberg J.B., Brustad N., Eliasen A., Mohammadzadeh P., Følsgaard N. (2024). High-dose vitamin D3 supplementation in pregnancy and risk of neurodevelopmental disorders in the children at age 10: A randomized clinical trial. Am. J. Clin. Nutr..

[18] Bandini L., Vinceti B., Urbano T., Plazzi G., Filippini T., Vinceti M. (2026). The impact of maternal vitamin D levels during pregnancy and risk of autism spectrum disorder and attention deficit hyperactivity disorder diagnosis and symptoms in offspring: A systematic review and dose-response meta-analysis. Eur. Child Adolesc. Psychiatry.

[19] Avram O.E., Bratu E.A., Curis C., Moroianu L.A., Drima E. (2025). Modifiable nutritional biomarkers in autism spectrum disorder: A systematic review and meta-analysis of vitamin D, B12, and homocysteine exposure spanning prenatal development through late adolescence. Int. J. Mol. Sci..

[20] Karras S.N., Shah I., Petroczi A., Goulis D.G., Bili H., Papadopoulou F., Harizopoulou V., Tarlatzis B.C., Naughton D.P. (2013). An observational study reveals that neonatal vitamin D is primarily determined by maternal contributions: Implications of a new assay on the roles of vitamin D forms. Nutr. J..

[21] Singh R.J., Taylor R.L., Reddy G.S., Grebe S.K.G. (2006). C-3 epimers can account for a significant proportion of total circulating 25-hydroxyvitamin D in infants, complicating accurate measurement and interpretation. J. Clin. Endocrinol. Metab..

[22] Bailey D., Perumal N., Yazdanpanah M., Al Mahmud A., Baqui A.H., Adeli K., Roth D.E. (2014). Maternal–fetal–infant dynamics of the C3-epimer of 25-hydroxyvitamin D. Clin. Biochem..

[23] Ooms N., van Daal H., Beijers A.M., Gerrits G.P.J.M., Semmekrot B.A., van den Ouweland J.M.W. (2016). Time-course analysis of 3-epi-25-hydroxyvitamin D3 shows markedly elevated levels in early life, particularly from vitamin D supplementation in preterm infants. Pediatr. Res..

[24] Mao D., Yuen L.Y., Ho C.S., Wang C.C. (2022). Maternal and neonatal 3-epi-25-hydroxyvitamin D concentration and factors influencing their concentrations. J. Endocr. Soc..

[25] Aghajafari F., Field C.J., Rabi D., Kaplan B.J., A Maggiore J., O’Beirne M., A Hanley D., Eliasziw M., Dewey D., APrON Study Team (2016). Plasma 3-epi-25-hydroxycholecalciferol can alter the assessment of vitamin D status using the current reference ranges for pregnant women and their newborns. J. Nutr..

[26] Al-Zohily B., Al-Menhali A., Gariballa S., Haq A., Shah I. (2020). Epimers of vitamin D: A review. Int. J. Mol. Sci..

[27] Karras S.N., Wagner C.L., Castracane V.D. (2018). Understanding vitamin D metabolism in pregnancy: From physiology to pathophysiology and clinical outcomes. Metabolism.

[28] Park H., Wood M.R., Malysheva O.V., Jones S., Mehta S., Brannon P.M., A Caudill M. (2017). Placental vitamin D metabolism and its associations with circulating vitamin D metabolites in pregnant women. Am. J. Clin. Nutr..

[29] Attwood T. (1998). Asperger’s Syndrome: A Guide for Parents and Professionals.

[30] Benjamini Y., Hochberg Y. (1995). Controlling the false discovery rate: A practical and powerful approach to multiple testing. J. R. Stat. Soc. Ser. B Stat. Methodol..

[31] Karras S.N., Anagnostis P., Naughton D., Annweiler C., Petroczi A., Goulis D.G. (2015). Vitamin D during pregnancy: Why observational studies suggest deficiency and interventional studies show no improvement in clinical outcomes? A narrative review. J. Endocrinol. Investig..

[32] Pilz S., Zittermann A., Obeid R., Hahn A., Pludowski P., Trummer C., Lerchbaum E., Pérez-López F.R., Karras S.N., März W. (2018). The role of vitamin D in fertility and during pregnancy and lactation: A review of clinical data. Int. J. Environ. Res. Public Health.

[33] von Elm E., Altman D.G., Egger M., Pocock S.J., Gøtzsche P.C., Vandenbroucke J.P. (2007). The Strengthening the Reporting of Observational Studies in Epidemiology (STROBE) statement: Guidelines for reporting observational studies. Lancet.

